# Endoscopic removal of esophageal bezoars using a condom as a novel retrieval device

**DOI:** 10.1055/a-2777-4711

**Published:** 2026-01-28

**Authors:** Jieru Guo, Haowei He, Guang Song, Shasha Liang, Long Rui, Xun Li, Shuang Liu

**Affiliations:** 1 926 Hospital of Joint Logistics Support Force, PLA, Kaiyuan, China; 2 Department of Gastroenterology, The Hospital of 82nd Group Army PLA, Baoding, China

**Fig. 1:**
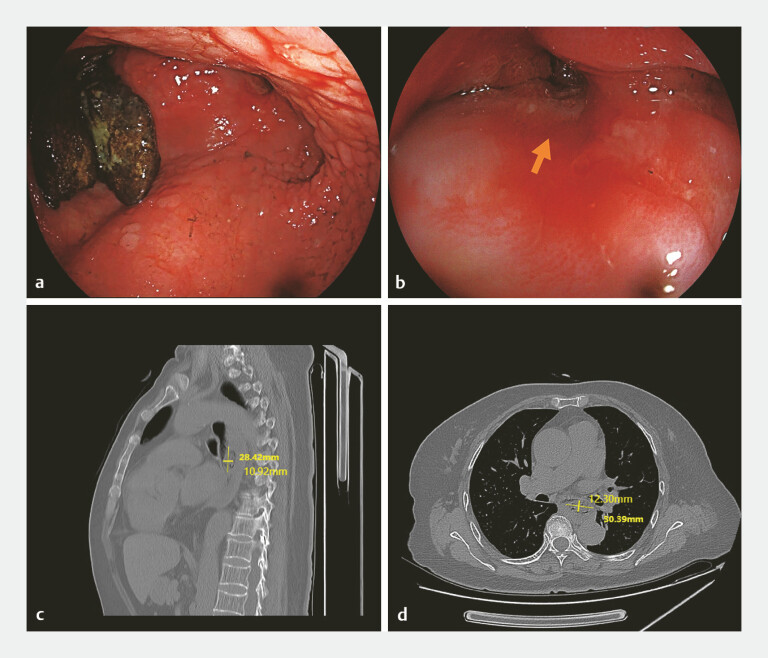
Pre-procedural localization of esophageal diospyrobezoars by endoscopy and CT. **a, b** Endoscopic views showing the diospyrobezoars with the adjacent esophageal ulcer (the arrow in b). **c, d** CT scans showing diospyrobezoars within the dilated esophageal lumen, located in close proximity to the left main bronchus and major blood vessels (outlined by the orange dashed line). CT, computed tomography.

A 64-year-old woman presented to our gastroenterology department with a 5-day history of dysphagia. She reported a history of persimmon ingestion and a 15-year history of achalasia. Gastrointestinal endoscopy revealed several diospyrobezoars retained within an epiphrenic diverticulum ( Fig. 1 **a** ). Esophagography identified an ulcer on the diverticulum wall near the cardia ( Fig. 1 **b** ). Computed tomography showed bezoars in the lower esophagus measuring 3.0 cm × 2.8 cm × 1.2 cm, associated with distal esophageal dilation ( Fig. 1 **c** ). Multiplanar reformation demonstrated the bezoarʼs proximity to the left main bronchus and major blood vessels ( Fig. 1 **d** ).

**Fig. 2:**
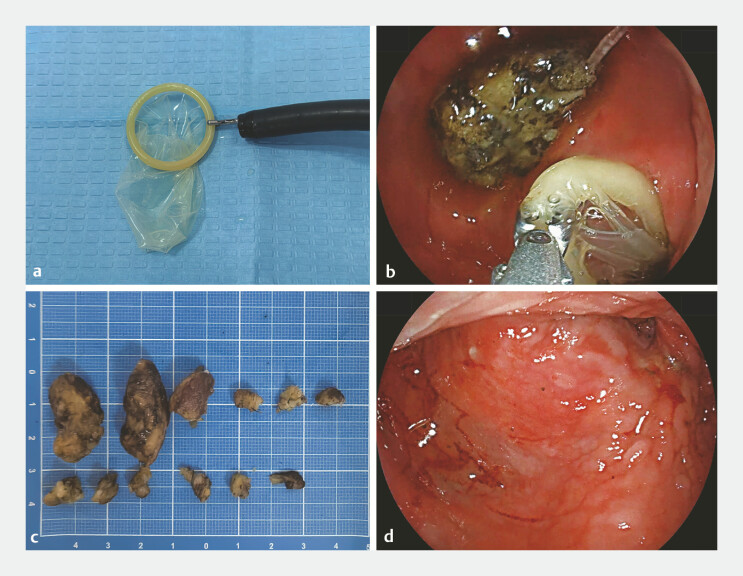
Endoscopic removal of esophageal diospyrobezoars using a condom as an improvised retrieval device. **a** A condom is grasped with foreign body forceps. **b** The condom is advanced to ensnare the diospyrobezoars. **c** All retrieved diospyrobezoars. **d** A post-procedure view confirming complete removal and mucosal integrity.

On the second day of admission, we performed the endoscopic bezoar removal. We employed a condom grasped by foreign body forceps as a retrieval bag ( Fig. 2 **a** ), advancing and repositioning it to entrap the diospyrobezoars ( Fig. 2 **b** ). After repeated attempts, all diospyrobezoars were successfully extracted ( Fig. 2 **c, d** ). The procedure was completed without complications, such as perforation and bleeding ( Video 1 ).

Endoscopic removal of esophageal diverticulum diospyrobezoars using an improvised condom device for entrapment and retrieval.Video 1

Postoperatively, the patient received adjunctive therapy with a proton pump inhibitor. At the 1-month follow-up, she was asymptomatic and report no chest pain or dysphagia.

Esophageal bezoars are even rarer than gastrointestinal bezoars. Their formation is strongly associated with esophageal structural and motility disorders, such as achalasia 1 . An esophageal bezoar can cause acute obstruction, gastrointestinal disturbances, ulceration, and even perforation 2 3 , as well as chest pain and dyspnea 4 . While retrieval baskets or snares are first-line interventions 5 , we innovatively utilized a condom as a retrieval device. Its soft and pliable nature makes it a strategically safer choice for complex retrievals, minimizing the risk of mucosal injury.

Endoscopy_UCTN_Code_TTT_1AO_2AL
